# High mechanical property silk produced by transgenic silkworms expressing the spidroins PySp1 and ASG1

**DOI:** 10.1038/s41598-021-00029-8

**Published:** 2021-10-25

**Authors:** Xiaoli Tang, Xiaogang Ye, Xiaoxiao Wang, Shuo Zhao, Meiyu Wu, Jinghua Ruan, Boxiong Zhong

**Affiliations:** College of Animal Science, Zhejiang University, Hangzhou, People’s Republic of China

**Keywords:** Biochemistry, Molecular biology

## Abstract

Spider silk is one of the best natural fibers with excellent mechanical properties; however, due to the visual awareness, biting behavior and territory consciousness of spiders, we cannot obtain spider silk by large-scale breeding. Silkworms have a spinning system similar to that of spiders, and the use of transgenic technology in *Bombyx mori*, which is an ideal reactor for producing spider silk, is routine. In this study, the piggyBac transposon technique was used to achieve specific expression of two putative spider silk genes in the posterior silk glands of silkworms: aggregate spider glue 1 (*ASG1*) of *Trichonephila clavipes* (approximately 1.2 kb) and two repetitive units of pyriform spidroin 1 (*PySp1*) of *Argiope argentata* (approximately 1.4 kb). Then, two reconstituted spider silk-producing strains, the AG and PA strains, were obtained. Finally, the toughness of the silk fiber was increased by up to 91.5% and the maximum stress was enhanced by 36.9% in PA, and the respective properties in AG were increased by 21.0% and 34.2%. In summary, these two spider genes significantly enhanced the mechanical properties of silk fiber, which can provide a basis for spidroin silk production.

## Introduction

The orb-weaving spider family, containing more than 25% of all living spider species, is one of the most diverse spider families, with seven morphologically differentiated silk gland types^1,2^. Each gland secretes one or more unique spidroins to generate a specific structure of spider silk fibers or jelly to form a protective shell, support the web structure, support the reproduction of offspring and acquire prey food^3^. Therefore, every spider silk has unique material properties, including viscosity, strength, hardness and extensibility. For example, dragline silk is the strongest fiber, approximately threefold tougher than aramid fibers and fivefold stronger than steel^4^. Due to its excellent mechanical properties, spider silk has been studied extensively.

Pyriform silk and aggregate silk are two of the seven spider silks on the orb web^5^. Pyriform silk secreted by the pyriform gland is generally mixed with dragline silk to form a textured composite filament, not a monofilament, called an attachment disc^6^. Generally, spidroins are composed of many specific repetitive units or motifs with large molecular weights, and these motifs are considered the basis for the unique mechanical properties of spider silk. The major component of pyriform silk is a highly repetitive protein; according to previous research, the complete cDNA sequence of pyriform spidroin 1 (*PySp1*) of *A. argentata* contains 21 complete repeats^7^. Unlike the main motifs of other spidroins (MiSp, MaSp, Flag), which are GPGGX, An, GA, or GGX and GPG^8^, the repeat motifs in the primary structure of pyriform spidroin are PXPXPX and QQSSVAQS^7,9,10^. The model peptide studies have shown that the proline-rich motifs promote elastomeric properties^11^. The aggregate glands exist in pairs, and are known for their sticky aqueous secretions—aggregate silk glue^12^. Aggregate silk glue improves the extensibility of the capture threads^13^. Previous studies have identified the sequence of aggregate silk glue genes (*ASG*1 and *ASG*2) of the golden orb-weaving spider *T. clavipes*^14^ and reported that there are no repetitive fragments and that the motifs are NVNVN and QPGSG^15^, which also differ from those of MiSp, MaSp and Flag. These different motifs contribute to the specific properties of pyriform and aggregate silk.

Since realizing the potential of spider silk, researchers have been working on producing synthetic spider silk, trying to express spider silk genes in various systems, including *Escherichia coli*^16^, yeast^17^, tobacco^18,19^, and mammalian cell culture systems^20,21^; however, the protein obtained needs a completely artificial folding process under harsh conditions. Some researchers have also sprayed minerals, such as carbon nanotubes^22,23^, grapheme^23^, silver nanoparticles^24^, and ion precursors^25^, on the surface of mulberry leaves to feed silkworms and have even forced spiders to spin silk artificially^26^. Consequently, the strength or toughness of silk fibers obtained by these methods was improved; however, for large-scale production, these methods are time-consuming and expensive.

Silkworm (*Bombyx mori*) silk is a highly utilized classic natural protein fiber with good biocompatibility, degradability, and flexibility and a high yield^27–29^. Although spiders and silkworms are not closely related in evolution, their characteristics of silk production are common in some ways. For instance, silkworms have a spinning system similar to that of spiders, and both spiders and silkworms can produce large amounts of soluble silk protein with high repeatability^30^; the silk production is achieved under mild conditions^31^; as well as the silk protein, stored in the form of a highly concentrated liquid crystalline solution, is then passed through a narrow duct to assemble into nanofibrils^32^; thus, silkworms are natural reactors for the production of reconstituted spider silk. Although territorialism and cannibalism preclude spider farming as a viable manufacturing approach, silkworms can be cultivated on a large scale. With the upgrading of gene editing technology, especially *piggy*Bac transposon technology, a new era of using transgenic technology to produce spider silk has begun. Studies have revealed that the overall mechanical properties of composite silk fibers improve as the re-MaSp1 chain length increases^33^, and the length and components of repeat motifs in spider silk play an important role in improving the mechanical properties of transgenic silk fibers^34–36^.

In this study, we generated two transgenic silkworm strains, PA and AG, which specifically expressed the 1.4 kb pyriform spidroin gene 1 (*PySp*1) of *A. argentata* and the 1.2 kb aggregate silk glue1 (*ASG*1) of *T. clavipes*, respectively, in the posterior silk glands (PSGs) of *Bombyx mori* by *piggy*Bac transposon technology. Based on our research results, these two unique spidroins have great potential for improving mechanical properties.

## Materials and methods

### Animals

In this experiment, the polymorphic diapause strain Lan 10 (maintained in the *Bombyx mori* genetics and breeding laboratory of Zhejiang University) was used as the experimental system, and fresh mulberry leaves were raised and fed in accordance with the standard feeding conditions (25 °C, 80% relative humidity) three times a day.

### piggyBac vector construction

We designed a series of plasmids separately including 2 repeats of *PySp*1 (1.4 kb, KY398016) derived from the orb-weaving spider *A. argentata*^7^ and 1 repeat of *ASG*1 (1.2 kb, EU780014) derived from *T. clavipes*^14^. To ensure high expression of spider genes in the transgenic silkworms, the repetitive unit sequences of *PySp*1 and *ASG*1 were optimized according to codon usage of the Fib heavy (FibH) chain in *Bombyx mori*^37^. Then, these spider silk genes (Supplementary Figs. S1 and S2) were synthesized (GenScript, China) and subcloned into pUC57 (TaKaRa, China) to generate the intermediate vectors pUC-2xPA, and pUC-1xASG1, which carried NheI and AgeI restriction sites. By digestion with these two restriction enzymes, the targeted spidroin fragments of 2 × *PySp*1 or 1 × *ASG*1 were inserted into the vector pBac[IE1-EGFP] (which was constructed and maintained in our laboratory) to produce the FibH-spider silk donor vectors pBac[IE1-EGFP]-2 × *PySp*1-A.arg and pBac[IE1-EGFP]-1 × *ASG*1-N.cla.

### Silkworm transformation

The donor and helper (encoded the transposase, which is the critical sequence of the piggyBac system) vectors were prepared as previously described with some modifications^38^. Then, the eggs were injected with donor and helper DNA mixtures at a final concentration of 400 ng/µl. Two groups were independently microinjected: (1) the PA group, injected with pBac[IE1-EGFP]-*PySp*1-2 × A.arg and the helper vector; and (2) the AG group, transformed with pBac[IE1-EGFP]-1 × *ASG*1-N.cla and the helper vector. Finally, these G0 eggs were reared under standard conditions (25 °C, 80% R.H.) to the moth stage; then, every moth was mated with a wild-type moth to produce the G1 generation. Positive transgenic individuals in the G1 brood were screened for EGFP expression in the body using a fluorescence microscope (Olympus SZX16, Japan). Each positive individual in the brood was reared to the moth stage and mated with wild-type moths to produce the G2 generation for the selection of stable transgenic silkworms.

### Inverse PCR analysis

Inverse PCR was carried out to analyze the spidroin gene insertion site in the positive silkworms according to a previous study^39^. Genomic DNA was extracted from the EGFP-positive PSGs of the G2 PA and G2 AG groups. Subsequently, the DNA was digested with Sau3AI and was then circularized by T4 DNA ligase (TaKaRa, China). PCR amplification was carried out using the circularized fragments as templates under standard conditions with primers designed based on the left arm of the piggyBac transposable element—L1-F and L1-R for the first PCR and L2-F and L2-R for the second PCR (Supplementary Table S1). The PCR-amplified fragments were sequenced after cloning into the pClone007 Blunt vector (TSINKGE, No. TSV-007B). The sequencing data were analyzed using the silkworm genome database (http://sgp.dna.affrc.go.jp/KAIKObase/) to analyze the precise location in the chromosome.

### Quantitative real-time PCR analysis

Quantitative real-time PCR analysis (qRT-PCR) was performed to analyze transcripts in transgenic strains^40^. The relevant primers were designed using Premier 5.0 (Supplementary Table S2). Total RNA samples were extracted from PSGs and middle silk glands (MSGs) using TRIzol Reagent (Invitrogen, USA) on the last day of the fifth instar. cDNA was then synthesized using a PrimeScript RT Reagent Kit with gDNA Eraser (TaKaRa, China). qRT-PCR was performed and expression detected in real time using a CFX96 Real-Time PCR Detection System (BIO-RAD, USA). The reaction was performed for 40 cycles in a 20.0 µl reaction mixture containing 12.5 μl of TB Green Premix Ex Taq (TaKaRa, China), 2 μl of cDNA template, and 1 μl of each PCR primer (10 μM). BmRp49 was selected as the endogenous control for qPCR analysis. To calculate the relative expression levels of the detected genes, a relative quantitative method (threshold cycle [ΔΔCt]) was used. All samples were analyzed in three independent replicates.

### Western blotting

Extraction of proteins from the cocoons of each transgenic silkworm strain was performed as previously described^41^. Briefly, 1 undegummed cocoon was selected randomly and cut into pieces, grinding with liquid nitrogen until to powder. 20-mg cocoon samples were suspended in 400 μl of SDS-protein extraction solution for 3 h at room temperature. Subsequently, the protein-containing supernatant of each sample was collected by centrifugation at 15,000 rpm for 10 min at 4 °C and diluted with protein loading buffer for further assays. The cocoon protein samples were loaded at equal volume into 4–15% gradient SDS-PAGE gels (Sangon, Shanghai). Proteins were visualized by staining with Coomassie brilliant blue R-250 (Sangon). Proteins extracted from the cocoons were transferred onto a PVDF membrane (Immobilon-P, Millipore) after separation by SDS-PAGE. The membrane was blocked with 3% BSA in TBS-T (10 mM Tris, 150 mM NaCl, and 0.1% Tween 20) and was then incubated with an anti-His antibody (1:5000 dilution, Sangon) as the primary antibody and peroxidase conjugate goat anti-rabbit IgG-HRP (1:5000 dilution, Sangon) as the secondary antibody. Signal detection was performed using a high-sensitivity ECL luminescence reagent kit (Sangon, Shanghai).

### Field emission scanning electron microscopy of the composite silk fibres

SEM analysis of the fibers was performed using a field emission scanning electron microscope (SU8010, Hitachi, Japan). The cross sections of the fibres were obtained after brittle fracturing in liquid nitrogen. The surface and cross sections of the fibres were placed on scanning electron microscopy stubs, and the fibers were observed and photographed after being coated with platinum using an ion sputtering instrument (MC1000, Hitachi) at an accelerating voltage of 2 kV for two minutes.

### Mechanical testing of transgenic silk fibers

Mechanical testing was performed as described in a previous study with some modifications^33^. Ten similarly shaped cocoons from each transgenic and control (non-transgenic) strain were randomly selected and treated as follows: each cocoon was first bathed in 100 °C deionized water for 2 min with slight shaking occasionally to remove the air inside the cocoon. The cocoon was then soaked in 65 °C water for 3 min to saturate all of the cocoon layers and immersed in deionized water at 100 °C for 2 min to soften the sericin. The cocoon was subsequently transferred to water at 85 °C for 15 min to remove the sericin. Finally, the cocoon was transferred to 70 °C deionized water, and five different parts of the silk fibers were obtained by manual drawing. Subsequently, 5 filaments from different parts of every cocoon were used for mechanical property measurements. The cross-sectional diameter of each two-brins silk sample was measured using a digital microscope (Keyence, Japan) at 1000× magnification; five measurements were obtained from each sample, and the average diameter was calculated, then obtained the two-brins cross-sectional area (S = ∏r^2^). Because the measured diameters may include the thin sericin layer, the cross-sectional areas were overestimated compared with the real silk fibre. Thus, the values for the mechanical properties might have been underestimated relative to the real silk fibres. The mechanical test were performed under ambient conditions using an AGS-J Universal Test instrument (Shimadzu Ltd., Japan) equipped with a 5 N load cell at a constant speed of 2 mm/min and frequency of 250 MHz. The load–displacement data sets were recorded automatically with the control software (Trapezium2, Shimadzu).

### Statistical analyses

Statistical analysis was performed using a two-tailed Student's t test to determine whether the averages were significantly different among the transgenic silkworm strains. A *P* value of < 0.05 was considered significant.

## Results

### piggyBac vector design and construction

**Figure 1:**
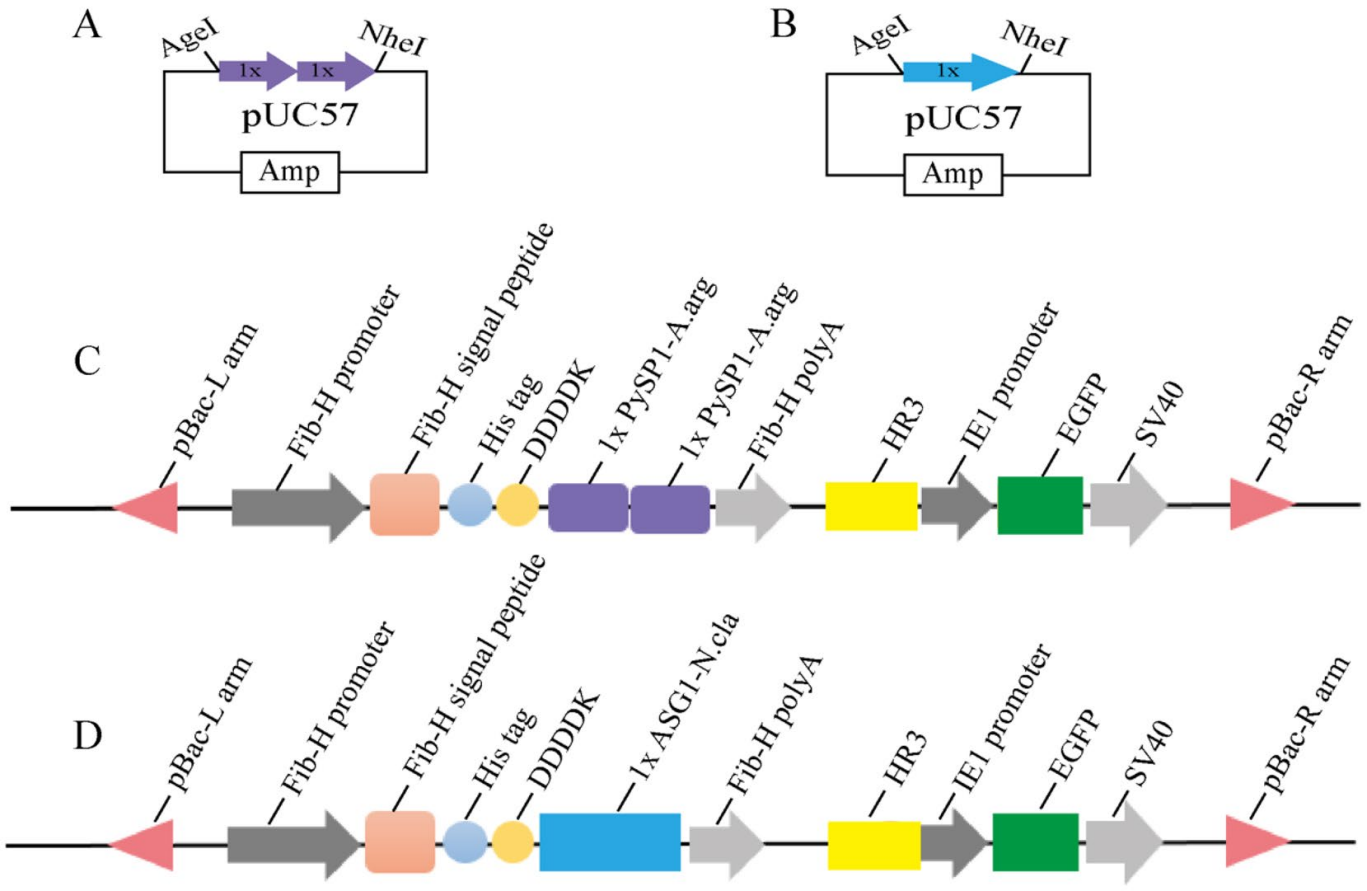
Structures of the intermediate vectors and donor vectors used in this study. (**A**,**B**) The intermediate vectors pUC-2xPA and pUC-1xASG1 carried AgeI and NheI, respectively. (**C**,**D**) Donor vectors pBac[IE1-EGFP]-2 × *PySp*1-A.arg and pBac[IE1-EGFP]-1 × *ASG*1-N.cla, respectively. The key elements are shown in different colors: purple represents one repeat of *PySp*1 of *A. argentata* (234 aa), and blue shows one repeat of *ASG*1 of *T. clavipes* (384 aa).

Synthetic gene modules of the two repetitive units of spider pyriform spidroin gene 1 (*PySp*1) of *A. argentata* and one repeat motif of aggregate silk glue1 (*ASG*1) of *T. clavipes* (Fig. 1A,B) were subcloned into the plasmid pBac[IE1-EGFP], which was constructed and maintained in our laboratory, to generate the FibH-spider silk donor vectors pBac[IE1-EGFP]-2 × *PySp*1-A.arg and pBac[IE1-EGFP]-1 × *ASG*1-N.cla (Fig. 1C,D). The key expression boxes included the polyA sequence of the Fibroin-heavy chain (Fib-H) gene, which was under the control of a Fib-H promoter sequence; two repeats of *PySp*1 (1.4 kb, Supplementary Fig. S1) or one repeat of *ASG*1 (1.2 kb, Supplementary Fig. S2), whose protein MWs were predicted to be 50 kDa and 45 kDa, respectively; and enhanced green fluorescence protein (EGFP) as the marker gene for screening positive individuals, which was induced by the IE1 promoter for nonspecific expression in the whole body.

### Silkworm transformation

**Figure 2:**
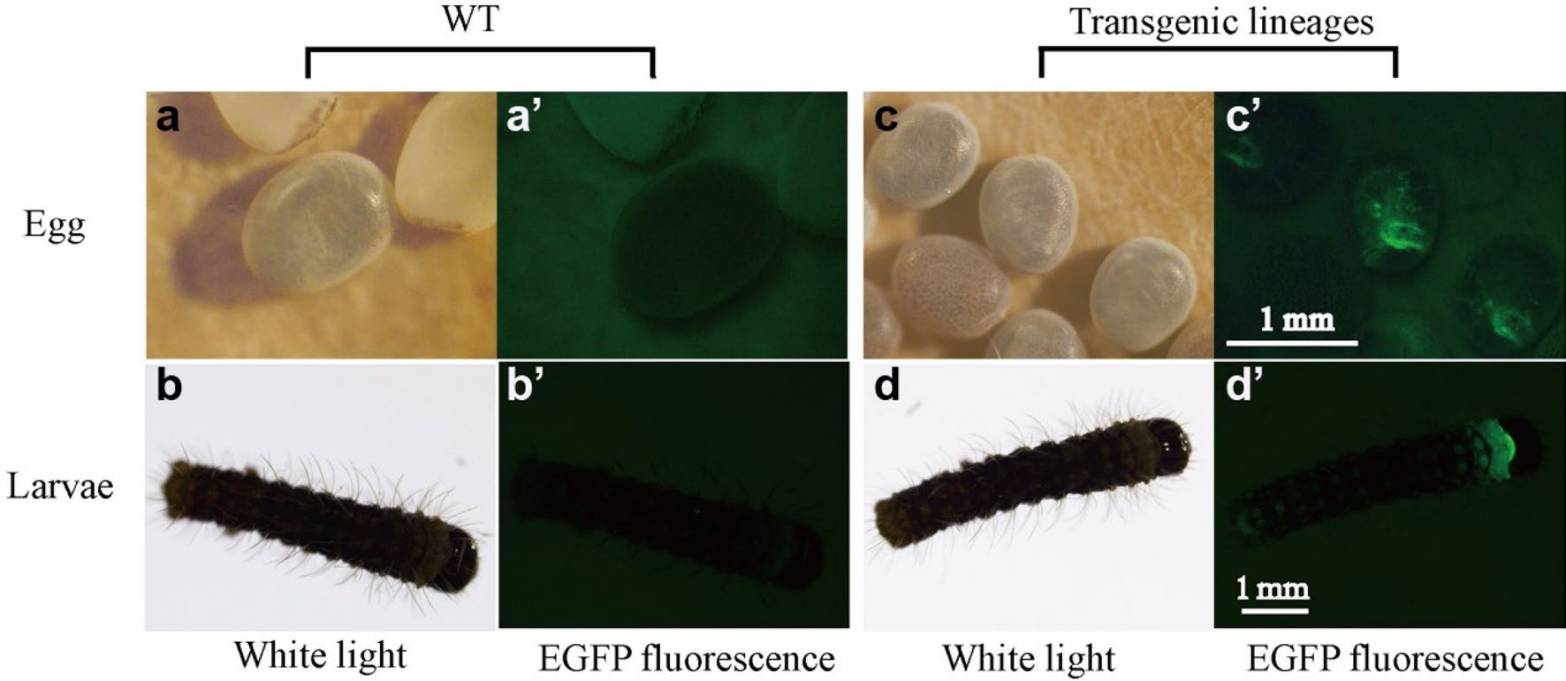
Positive transgenic strains for EGFP-specific expression in the G1 stage. (**a**,**a′**) G1 eggs and (**b**,**b′**) larvae of the WT strains were viewed under white light and EGFP fluorescence microscopy, respectively; (**c**,**c′**) G1 eggs and (**d**,**d′**) larvae of the positive strains were viewed under white light and EGFP fluorescence microscopy, respectively. The phenotypes of these two transgenic strains were similar under green fluorescence microscopy.

In total, 600 eggs of the Lan10 strain were microinjected with a mixture of the donor and helper vectors. After injection, the embryos (G0) were reared to the larval stage to hybridize to produce the G1 brood. The positive G1 transgenic silkworms were screened for the green fluorescence signal in the body (Fig. 2). The data of the transgenic silkworms are shown in Table 1; 80% of G0 embryos hatched to larvae and were then hybridized to produce the G1 brood. Consequently, we separated 1 (3.84%) positive G1 individual of the PA strain and 1 (2.44%) positive individual of the AG strain. To obtain more stable transgenic strains, the G1 PA and AG moths were mated with wild-type moths to produce the G2 generation (PA; AG).

**Table 1:** Microinjection into embryos of the Lan10 strain.

Transgenic system	Transgenic strain	Injected concentration (ng/µl)	Injected embryos (G0)	Hatched embryos (G0)	Hatched (%)	G1 broods	Positive broods (G1)	G1 positive (%)
pBacPySp1x2-A.arg + helper	PA	400	600	443	73.83	26	1	3.85
pBacASG1x1-N.cla + helper	AG	400	600	508	84.67	41	1	2.44

### Inverse PCR analysis

**Figure 3:**
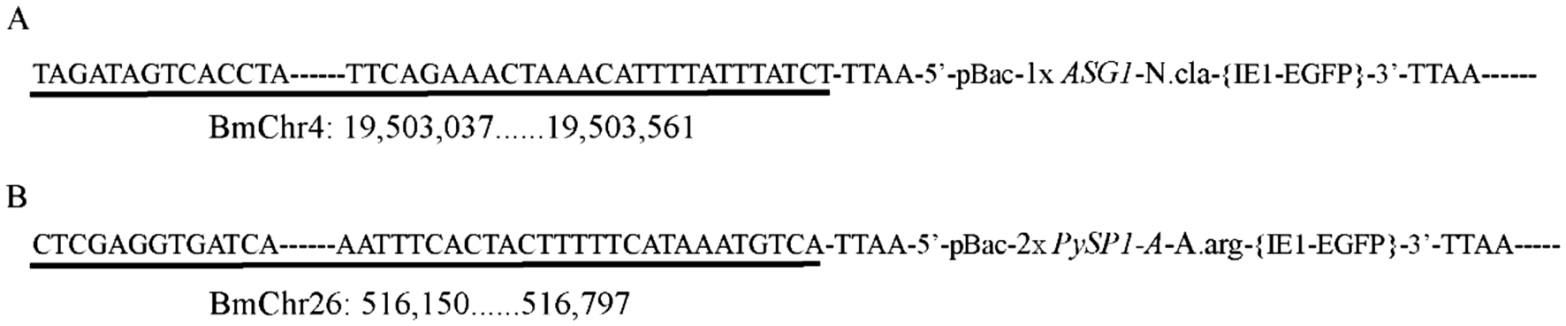
The genomic insertion site in the transgenic strain. (**A**) The genomic insertion site in the AG strain, BmChr.4; (**B**) The genomic insertion site in the PA strain, BmChr.26. Both the AG and PA strains exhibited a single insertion.

To analyze the precise insertion site in the obtained transgenic strain and identify the effect of the position on the expression of the spidroins, genomic DNA was extracted from the PSGs of the G2 EGFP-positive individuals, digested with Sau3AI and confirmed by inverse PCR. Only one precise integration site of these two donor vector was detected respectively, which was chromosome 4 in the AG strain (Fig. 3A, Supplementary Fig. S3), and chromosome 26 in the PA strain (Fig. 3B, Supplementary Fig. S4). The different insertion sites of these two spidroins also suggested that the *piggy*Bac transposon was inserted randomly, similar to observations in previous studies^42–44^.

### Quantitative real-time PCR (qRT-PCR) analysis

**Figure 4:**
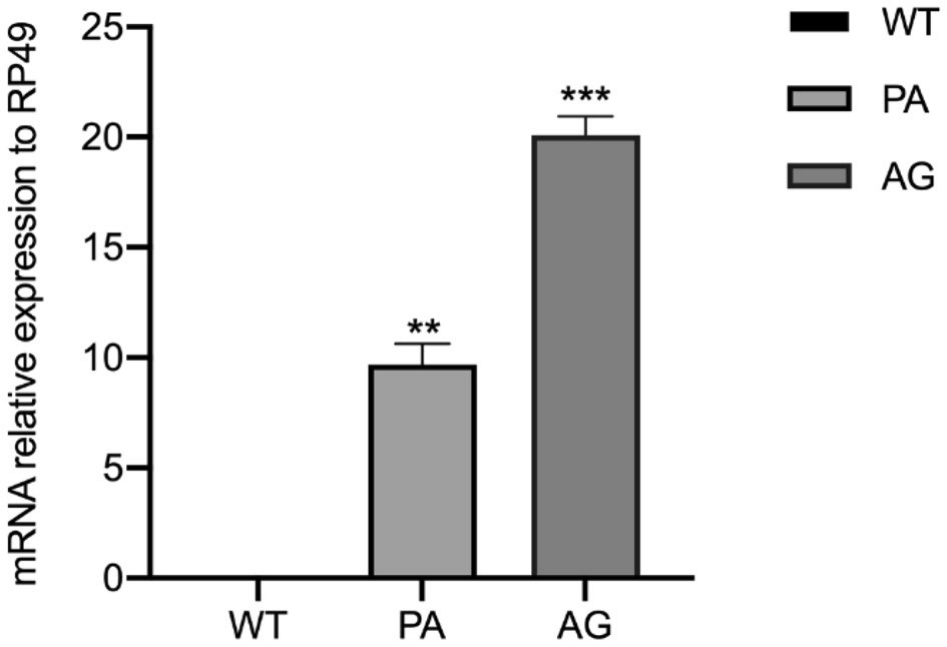
Comparative analysis of spidroin gene expression in the PSGs of transgenic strains on the last day of the fifth instar. The mean ± SD values were derived from three biological replicate experiments. The significance of the difference between transgenic strains (PA; AG) and WT was calculated using two-tailed Student’s t-tests. *P < 0.05; **P < 0.01; ***P < 0.001.

The relative mRNA levels of *PySp1* and *ASG1* in the PSGs and MSGs on the last day of the 5th instar of the G2 WT, PA and AG strains were analyzed using qRT-PCR. Comparison showed that the signal was detected in PSGs of the transgenic strains but not the WT strain or MSGs (Fig. 4, Supplementary Fig. S5), indicating that the spidroin genes were integrated into the silkworm genome and stably inherited; the expression levels of *PySp1* and *ASG1* showed significant differences among the different transgenic strains (Fig. 4). The transcript level of AG was the highest and was twofold higher than that in PA (Fig. 4). Based on analysis of the length of spidroin genes, we speculated that the integration of larger exogenous genes can induce lower transcript expression levels.

### Western blotting for transgenic silkworm proteins

**Figure 5:**
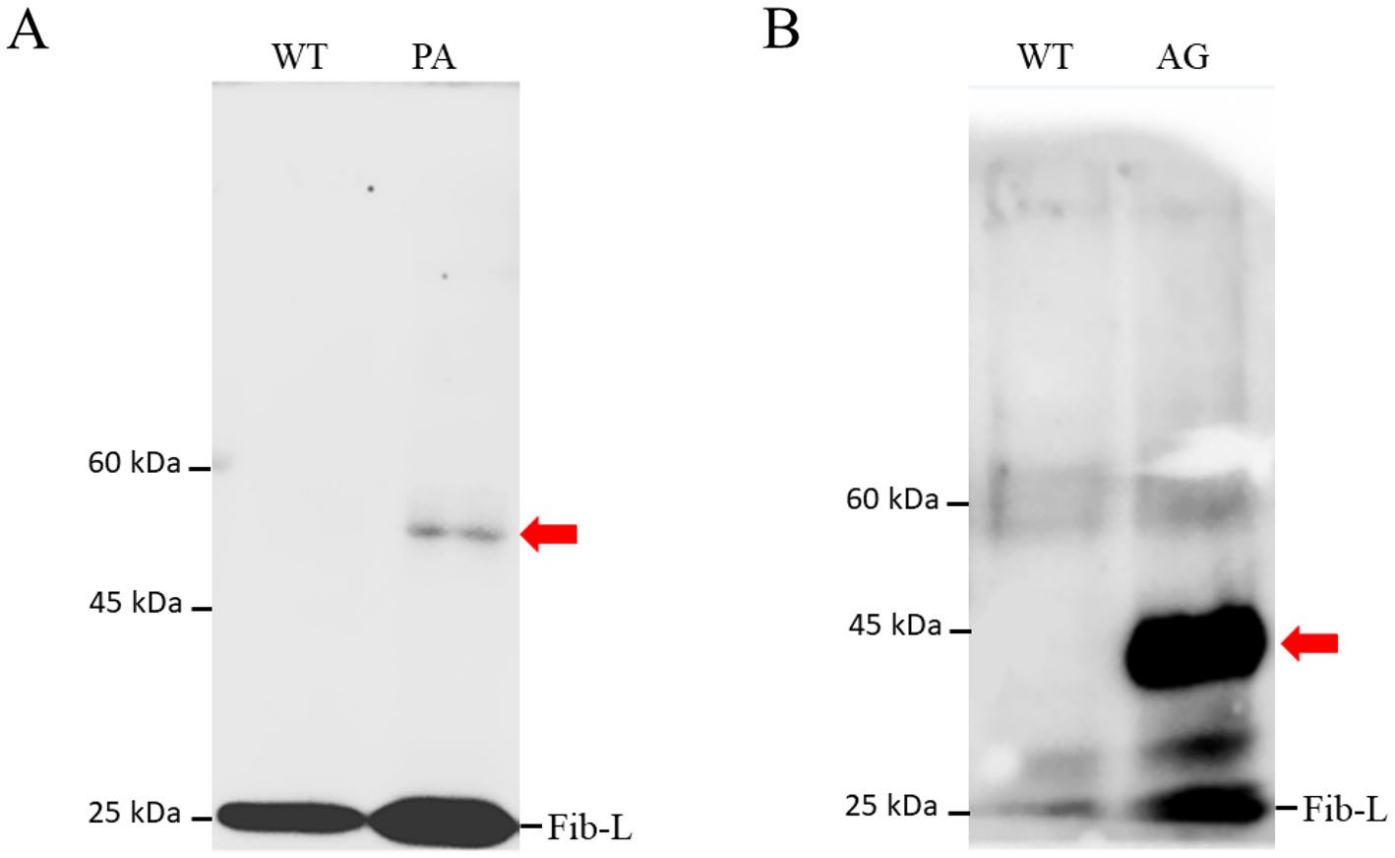
Western blot analysis of spidroin proteins extracted from cocoons. (**A**) Western blot analysis of cocoons of the G2 PA strains (50 kDa). (**B**) Western blot analysis of cocoons of the G2 AG strain (45 kDa). The red arrows indicate the predicted bands. Fibroin light chain (Fib-L), non-specific bands. The original figures are shown in Supplementary Fig. S7.

The proteins extracted from the cocoons were subjected to SDS-PAGE and Western blot analyses to further investigate *PySp*1 and *ASG*1 expression at the translational level. For the image of SDS-PAGE, it is impossible to distinguish the bands corresponding to the proteins of interest because of too many weak bands near the target area (Supplementary Fig. S6). As for the western blotting, a single band with the predicted size for each protein (*PySp*1, 50 kDa; *ASG*1, 45 kDa) was detected in cocoons of both the G2 PA and G2 AG strains (Fig. 5, Supplementary Fig. S7), suggesting that *PySp*1 and *ASG*1 were successfully secreted into the transgenic silkworm cocoons and formed a reconstituted spider-silk fiber. As for the relative expression level of these two proteins in cocoons, may be related to mRNA expression level and the structure of spidroin gene.

### Mechanical testing of transgenic silk fibers

**Figure 6:**
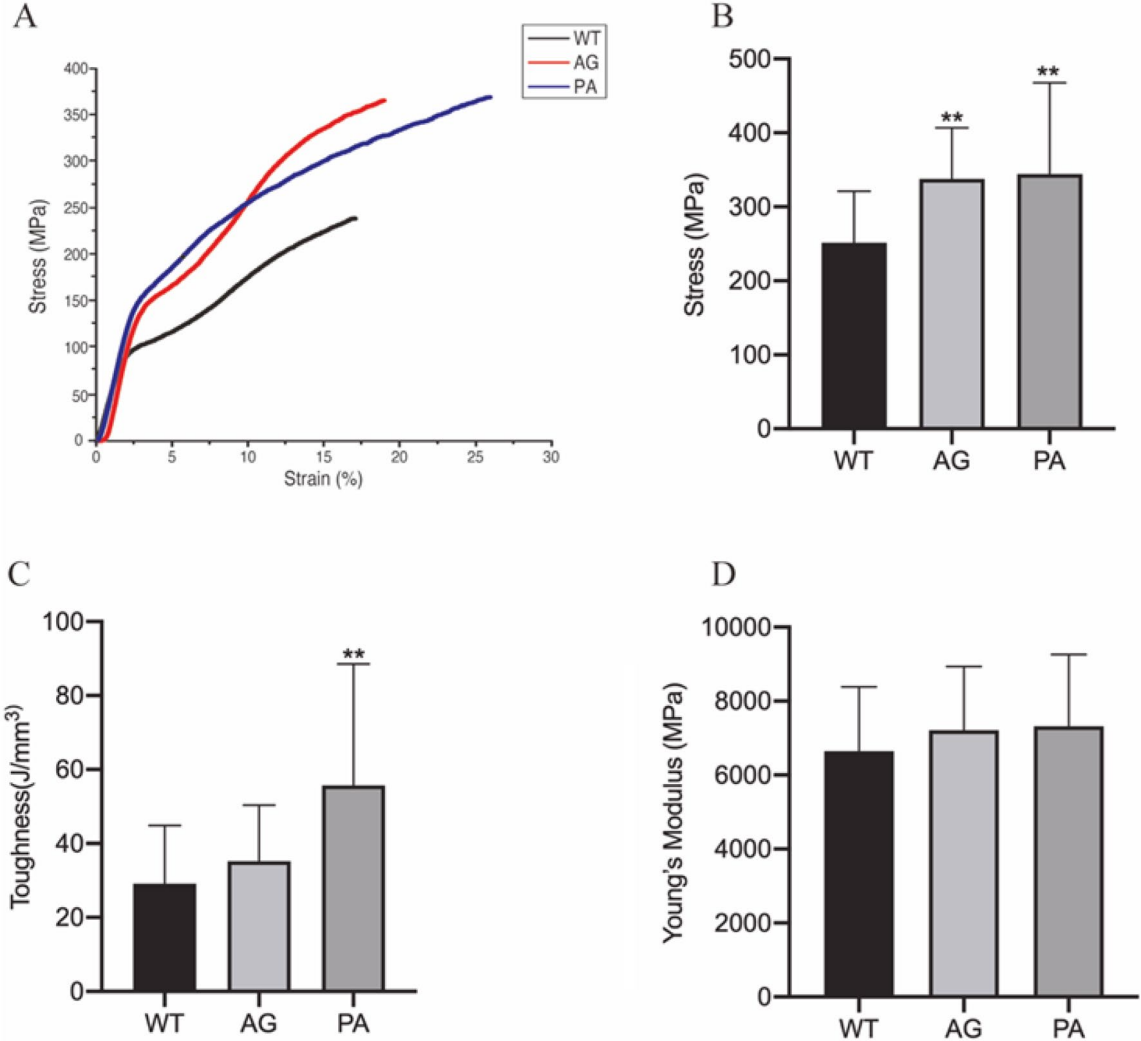
Mechanical properties of the recombined spider silk fibers from the PA and AG strains. (**A**) Stress–strain curves of WT and FibH-spider silk fibers, which were tested at a speed of 2 mm/min under equivalent conditions. The result for the WT fiber is shown in black, and the lines in red and blue indicate the results for the AG and PA strains, respectively. Fifty samples from each transgenic strain were tested. (**B**–**D**) Graphs of the maximum stress, toughness and Young’s modulus, respectively. The significance of the difference between transgenic fibers and WT was calculated using two-tailed Student’s t-tests. *P < 0.05; **P < 0.01.

To eliminate the influence of environmental differences on the comparability of silk strength, we selected composite silk and control silk from the same feeding season to ensure an accurate comparison. The fibers from transgenic and WT cocoons were randomly selected for scanning electron microscopy analysis of the fibre surface and cross section. The results showed that the morphology structure among WT, PA and AG exhibited no obvious differences (Supplementary Fig. S8). The mechanical properties of single two-wire composite fiber from transgenic and WT cocoons were measured under the same conditions. The tensile tests produced unimodal stress–strain curves despite using 2-brin fibers. Figure 6 shows that the mechanical properties of spider silk fibers, including the maximum stress, toughness and Young’s modulus, varied among the different transgenic strains but were generally higher than those of fibers from the control group. The maximum stress of the reconstituted silk fibers from the AG and PA strains was 34.2% and 36.9% higher than that of the WT fibers, respectively (Fig. 6B, Table 2); the toughness of the reconstituted silk fibers from the AG and PA strains was 21.0% and 91.5% higher than that of the WT fibers, respectively (Fig. 6C, Table 2); the Young’s modulus of the reconstituted silk fibers from the AG and PA strains was 6.3% and 7.9% higher than that of the WT fibers, respectively (Fig. 6D, Table 2). Among these two transgenic strains, the PA strain showed better properties, and we speculated the difference of mechanical properties maybe caused by the different motifs in spidroin, which similar to findings of previous studies^36^.

**Table 2:** Mechanical properties of the Spider Silk Fibers in spider silk-producing PA and AG strains. Ten similarly shaped cocoons from each transgenic and control (non-transgenic) strain were randomly selected, and 5 filaments from different parts of every cocoon were used to measure the mechanical properties at a speed of 2 mm/min. The average and SD values were derived from fifty biological replicate experiments.

	WT		AG			PA		
	Ave.	SD	Ave.	SD	%	Ave.	SD	%
Maximum stress (MPa)	251.55	69.51	337.57	69.43	34.2	344.34	123.33	36.9
Toughness (MJ/m^3^)	29.10	15.77	35.21	15.16	21.0	55.72	32.84	91.5
Young’s Modulus (MPa)	6784.35	1470.56	7211.92	1721.46	6.3	7318.93	1945.81	7.9

## Discussion

In this study, we reported a strategy to improve the mechanical properties of silk fibers by piggyBac technology, successfully expressing *A. argentata* pyriform spidroin 1 (*PySp*1, 1.4 kb) and *T. clavipes* aggregate spider glue 1 (*ASG*1, 1.2 kb) in the PSGs of *Bombyx mori.* Western blot analyses suggested that *PySp*1 and *ASG*1 were successfully secreted into transgenic silkworm cocoons and formed a recombined spider silk fiber. Table 2 shows that the mechanical properties of the recombined spider silk fibers were significantly superior to those in the control group, especially the toughness, which improved by 21.0% or 91.5%. The use of piggyBac technology to genetically modify silkworms has been previously reported, especially the use of the silk protein gene promoter to drive the expression of the chimeric spider silk gene, and the improved mechanical properties of transgenic fiber were confirmed^33,35^. However, in silkworms and other species, almost all studies focus on dragline silk^16,17^, and few experiments use other spider silk genes. Although great efforts have been made in research on dragline silk, it is necessary to introduce new ideas, such as the use of other spider silk proteins, with very different motifs from those of dragline silk proteins.

Previous studies reported the impact of both the size and motifs of transgenic spider silk protein on the mechanical properties of different transgenic silk fibers^33^. Proteins with different motifs vary in mechanical properties; MiSp and MaSp contain many GPGXX, GA, and GGX motifs, which contribute to the formation of β sheets and provide tensile strength^45^, the flagelliform silk (Flag) spun by Argiope trifasciata spiders shows remarkable tensile properties due to the dominant presence of the –GGX– and –GPG– motifs and polyglycine II nanocrystals in its sequence^8^, while the repeat motifs of *PySp*1 are PXPXPX and QQSSVAQS^46^. The proline-rich motifs are predicted to produce a random coil configuration that promotes elastomeric properties^46^. In *ASG*1, the common motifs are NVNVN and QPGSG^15^, which differ from those of MiSp and MaSp, whereas the recombined *PySp*1 silk and *ASG*1 silk showed a significant improvement in toughness. It has been reported that *ASG1* might not be a spidroin subtype at all, but instead a mucin-like matrix protein, which could nevertheless provide structural reinforcement^5^. In light of this, we hypothesize that the co-expression of structural proteins other than silk proteins could have a positive effect on the mechanical properties of silkworm silk. The repeating units in *ASG*1 are dominated by proline and threonine, and this repeating units are speculated to promote the mechanical properties of silk. Thus, we speculated that the various repetitive motifs were the primary molecular basis for the unique mechanical properties of various spider silks, while proteins with different repetitive motifs could be important for the outstanding mechanical properties of the reconstituted silk fibers despite the low expression levels of the PySp1 and ASG1 proteins. On the other hand, *Bombyx mori* is an excellent heterologous host for expressing recombinant spider silk protein. Several previous reports have confirmed that the fibers of the transgenic silkworms encoding the silkworm/spider silk proteins have excellent mechanical properties despite the extremely low expression of the spider silk proteins^33,35^, which indicates that the production of composite silk fibers containing stably integrated chimeric silkworm/spider silk proteins in the silkworm has enormous potential.

The mechanical properties of the composite fibers increased with increasing length of the exogenous spider gene, and there was a significant linear relationship between the mechanical properties and the length^34^. Moreover, the structure and number of various repeat motifs in silk genes play an important role in improving the mechanical properties of composite silk fibers^33^. A higher number of repeat motifs in the exogenous spider gene can increase the crystallinity of silk fibers, which is closely related to their mechanical properties. Thus, creating a strategy to produce spidroin with higher expression levels and larger molecules is of great importance in improving the mechanical properties of transgenic silk. According to our research, we postulate that it is worthwhile to further investigate whether the mechanical properties of reconstituted silk, such as the maximum stress, maximum strain and toughness, can be improved further by importing more repeats or motifs, even exceeding the length of the natural spider genes, into the silkworm genome.

In summary, two kinds of chimeric spider silk were obtained in this study, and their mechanical properties were significantly superior to those of wild-type silk. This study expanded the application of the spider silk gene and laid a foundation for obtaining composite silk with improved mechanical properties.

## Supplementary Information

Supplementary Information.
