# Supplementary material for: High mechanical property silk produced by transgenic silkworms expressing the spidroins PySp1 and ASG1
Source: Sci Rep. 2021 Oct 25;11:20980. doi: 10.1038/s41598-021-00029-8 (PMC8546084; doi:10.1038/s41598-021-00029-8)
Supplement: Supplementary file 1 — Supplementary Information. [file 41598_2021_29_MOESM1_ESM.docx]

**High Mechanical Property Silk Produced by Transgenic Silkworms Expressing the Spidroins PySp1 and ASG1**

Xiaoli Tang^1†^, Xiaogang Ye^1†^, Xiaoxiao Wang^1^, Shuo Zhao^1^, Meiyu Wu^1^, Jinghua Ruan^1^, Boxiong Zhong^1*^

^1^College of Animal Science, Zhejiang University, Hangzhou, P. R. China.

*Corresponding author. Tel/Fax: +86-571-86971302; Email: [bxzhong@zju.edu.cn](mailto:bxzhong@zju.edu.cn)

† Xiaoli Tang and Xiaogang Ye have contributed equally to this work.

This file includes:

**Supporting Information**

Supplementary Figures S1-S6

Supplementary Tables S1-S3


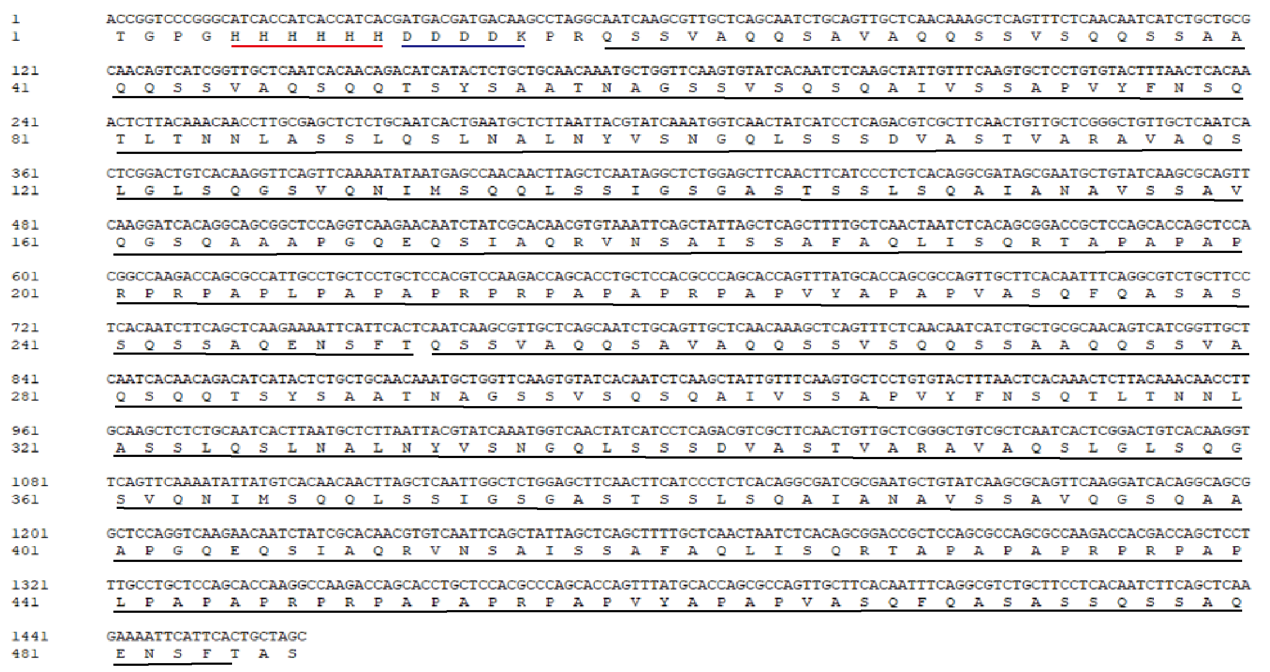


**Figure S1. The sequence of** **pUC-2xPA.** The regions underlined in red, blue and black indicate the sequence of the His tag, enterokinase and one repeat of *Argiope argentata* pyriform spidroin 1 (*PySp*1), respectively.


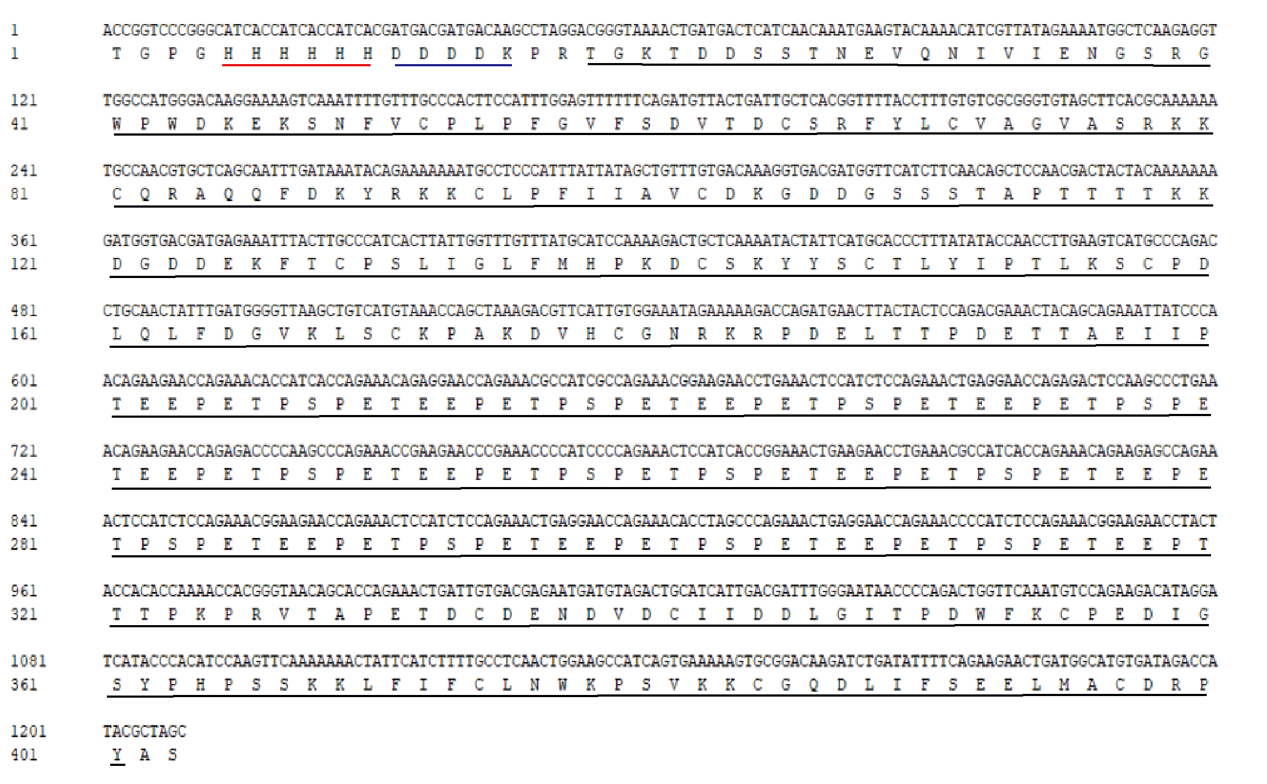


**Figure S2. The sequence of pUC-1xASG1.** The regions underlined in red, blue and black indicate the sequence of the His tag, enterokinase and one repeat of *Nephila clavipes* aggregate spider glue 1 (*ASG*1), respectively.

**Genome sequence (Bmchr4:19503740-19502860):**

5’TGGTTTAGAAGAGTACTGCTGGTTATTTTTAGTTGTATTTGCCTTTTGAATATCATTGATAAGGTTGAGAACCAGTGGTTTAGAATATATTATGTTTAATTTTGGATTCAGTGCTCTGTCAACACTCATTGCTGTACAATTATAGGATTATTTCGATGACAAGGCCTTTGAATAGATCTTAGATAGTCACCTTATATAGATATATTTTGAATATAATAACTATTAGGTAGTAATCTTGGTAATCACTTGAAATTGATGATATATTTGTATAAAACAGTTATAGGTATATTGAAATTGTTATCTGTAGGGCCAATGTGTATCTGTTCCATAAAACGTGTGTAAAGGCTCCATAAAAAACGAATTCGTAGCTTATAAGAAATATCCTAAAAAACTTCGTGTTAAAATTAATTCGAACGTGTTAAAAGGTTAACTCCGACCTTATCGGTATCGGCGTTAGCACCTTCGCTGTTCGGTTAGCAGTACGTGTTTACTTGGGACGGTTCAACTATTTCAAATATGCTGTAACTTTGTCCCCGAAAAATTATATATTTCTGTACTAAATATCTCTGCGATTTCATATTGTTATTATTTATATTAATTTTATTGTTAATATTACTACCGCTAGTGGGTTTAACTCCCACACACAACACTACAGCCTCAACACGCGAGGTTGAGAATTCAGAAACTAAACATTTTATTTATCTTTAATTATGAATCTATAGATTCGATCGCTTCGTTTGGATCAGAAAAAGACGCTGAAGACGTTCCTTTTAGCCACCCACTCGTTCCTTTTAGCTCTAGTTTGGTAGGCAGTGGCTTGGCTCTGCCTCTGGCATTGCTGACGTCCATGAGCCACAGTAACCACTTACCATCAGGTGGGC3’

**Sequence identified by inverse PCR:**

5’TGGGATGTTCTTTAGACGATGAGCATATCCTCTCTGCTCAGCTCGAGGTTAGATAGTCACCTTATATAGATATATTTTGAATATAATAACTATTAGGTAGTAATCTTGGTAATCACTTGAAATTGATGATATATTTGTATAAAACAGTTATAGGTATATTGAAATTGTTATCTGTAGGGCCAATGTGTATCTGTTCCATAAAACGTGTGTAAAGGCTCCATAAAAAACGAATTCGTAGCTTATAAGAAATATCCTAAAAAACTTCGTGTTAAAATTAATTCGAACGTGTTAAAAGGTTAACTCCGACCTTATCGGTATCGGCGTTAGCACCTTCGCTGTTCGGTTAGCAGTACGTGTTTACTTGGGACGGTTCAACTATTTCAAATATGCTGTAACTTTGTCCCCGAAAAATTATATATTTCTGTACTAAATATCTCTGCGATTTCATATTGTTATTATTTATATTAATTTTATTGTTAATATTACTACCGCTAGTGGGTTTAACTCCCACACACAACACTACAGCCTCAACACGCGAGGTTGAGAATTCAGAAACTAAACATTTTATTTATCTTTAACCCTAGAAAGATAGTCTGCGTAAAATTGACGCATGCATTCTTGAAATATTGCTCTCTCTTTCTAAATAGCGCGAATCCGTCGCTGTGCATTTAGGACATCTCAGTCGCCGCTTGGAGC3’

**Figure S3. Inverse PCR sequencing results of AG strain.** The matching nucleotide sequence is shown in red (Bmchr4:19503561~19503037). The blue letters indicate the arm sequence. The green letters indicate the inserted position.

**Genome sequence (Bmchr26: 516935~516001):**

5’GTACGTTCCAGCAGCACGCGCGCACCCTGCAGCTGGAGTGCGCGCCCGGCGACCCCGCCACGCTCGTCTGGACCGTCGCGCACGAGACGCCCGACCTGCTGTACTACCAGGTACGTTCGCTCACCACGCCTTAACATAGATCAACGTTACAGTCAAACGTCACAAATCTGAATGAATCGCTCAGACATTGCACTTTCGTTATAAGCAGCAAGGAATGTATATTATAAATTTACTTCAACAAAAATTCAATTAATCGAAAGCACACCATTTACTAATGTTTCTCTCACAGTGCTATACCCACAACAACCTAGGATGGAAAATCCACGTGGTGGACCCAGGTACCGCTGTCCCAAAACCCGGTGACCAAAAAGCCAGAATCAACGCAGCTCATAGGATTTGCCCATTTTCAAGCACGACATTCGTCTTAGTAATTACGTTACTTAATATGGCCGTCAGATAAAAACTGAATGGTTTGTAAGAGTTGGTGGTGTTGTCAGCATTGTGAAGCCTTTCAACGTTCGCTATGTTCAAAAATATCAAAATTAATGAAATATTCAGTTCTATTCATTCATATTATTAGTAATTTTGTAAATTAAAAAAAACAGCATCATCAACTCATCAAACTATTGTGATAGATATTATATAATTTTTGGGAATGAACAACAATCCAAGGGTATATATTGTCAATCTACGTACATTATCAATTCCATATCAAACAAGTATTAAATGATTCAGGAATTTTAACTATTTTACAGAATTAATTTCACTACTTTTTCATAAATGTCATTAAGGTGACTAGAAATTAAAATACTAATACTCACTTCAATGTTGAACAAAATATTAAACTACCTGACAAAAATAATAAATGAGGGTAGGTTAGTATTTTTGCAGATACAGGTGATTTTTCCTTATTTTTAATATGGCGGTATGTCAGC3’

**Sequence identified by inverse PCR:**

5’TGGGATGTTCTTTAGACGATGAGCATATCCTCTCTGCTCAGCTCGAGGTGATCAACGTTACAGTCAAACGTCACAAATCTGAATGAATCGCTCAGACATTGCACTTTCGTTATAAGCAGCAAGGAATGTATATTATAAATTTACTTCAACAAAAATTCAATTAATCGAAAGCACACCATTTACTAATGTTTCTCTCACAGTGCTATACCCACAACAACCTAGGATGGAAAATCCACGTGGTGGACCCAGGTACCGCTGTCCCAAAACCCGGTGACCAAAAAGCCAGAATCAACGCAGCTCATAGGATTTGCCCATTTTCAAGCACGACATTCGTCTTAGTAATTACGTTACTTAATATGGCCGTCAGATAAAAACTGAATGGTTTGTAAGAGTTGGTGGTGTTGTCAGCATTGTGAAGCCTTTCAACGTTCGCTATGTTCAAAAATATCAAAATTAATGAAATATTCAGTTCTATTCATTCATATTATTAGTAATTTTGTAAATTAAAAAAAACAGCATCATCAACTCATCAAACTATTGTGATAGATATTATATAATTTTTGGGAATGAACAACAATCCAAGGGTATATATTGTCAATCTACGTACATTATCAATTCCATATCAAACAAGTATTAAATGATTCAGGAATTTTAACTATTTTACAGAATTAATTTCACTACTTTTTCATAAATGTCATTAACCCTAGAAAGATAGTCTGCGTAAAATTGACGCATGCATTCTTGAAATATTGCTCTCTCTTTCTAAATAGCGCGAATCCGTCGCTGTGCATTTAGGACATCTCAGTCGCCGCTTGGAGC3’

**Figure S4. Inverse PCR sequencing results of PA strain.** The matching nucleotide sequence is shown in red (Bmchr26: 516797~516150). The blue letters indicate the arm sequence. The green letters indicate the inserted position.


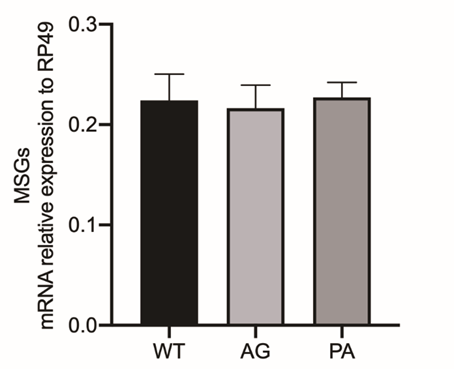


**Figure S5.** **Comparative analysis of spidroin gene expression in the MSGs of transgenic strains on the last day of the fifth instar.** The mean+SD values were derived from three biological replicate experiments. The significance of the difference between the PA and AG strains was calculated using two-tailed Student’s t-tests.


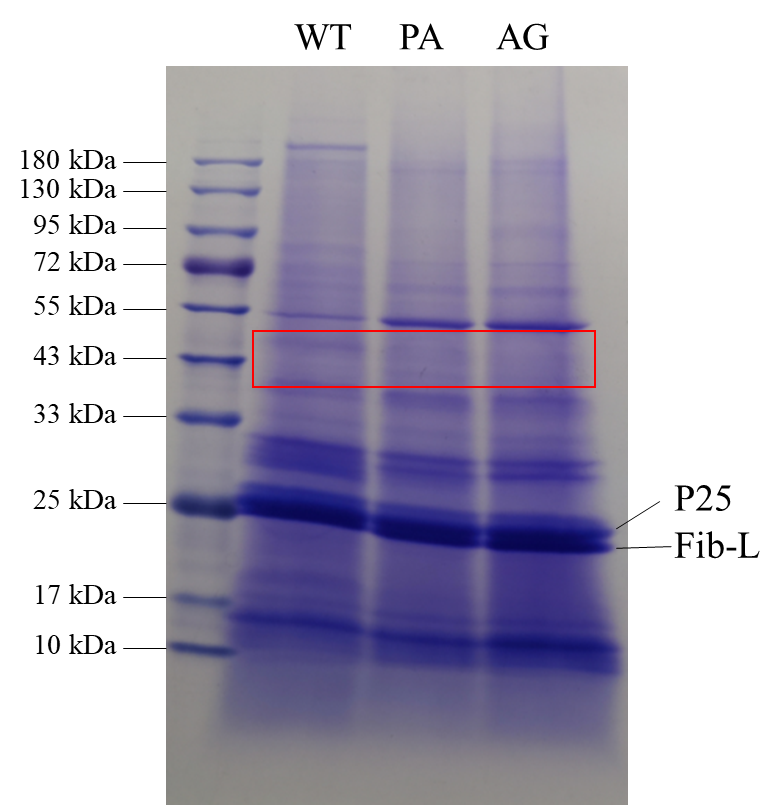


**Figure S6. The Image of SDS-PAGE.** The red rectangle indicates the predicted location of the target bands. Fib-L, fibroin light chain; P25, 25-kD polypeptide proteins.


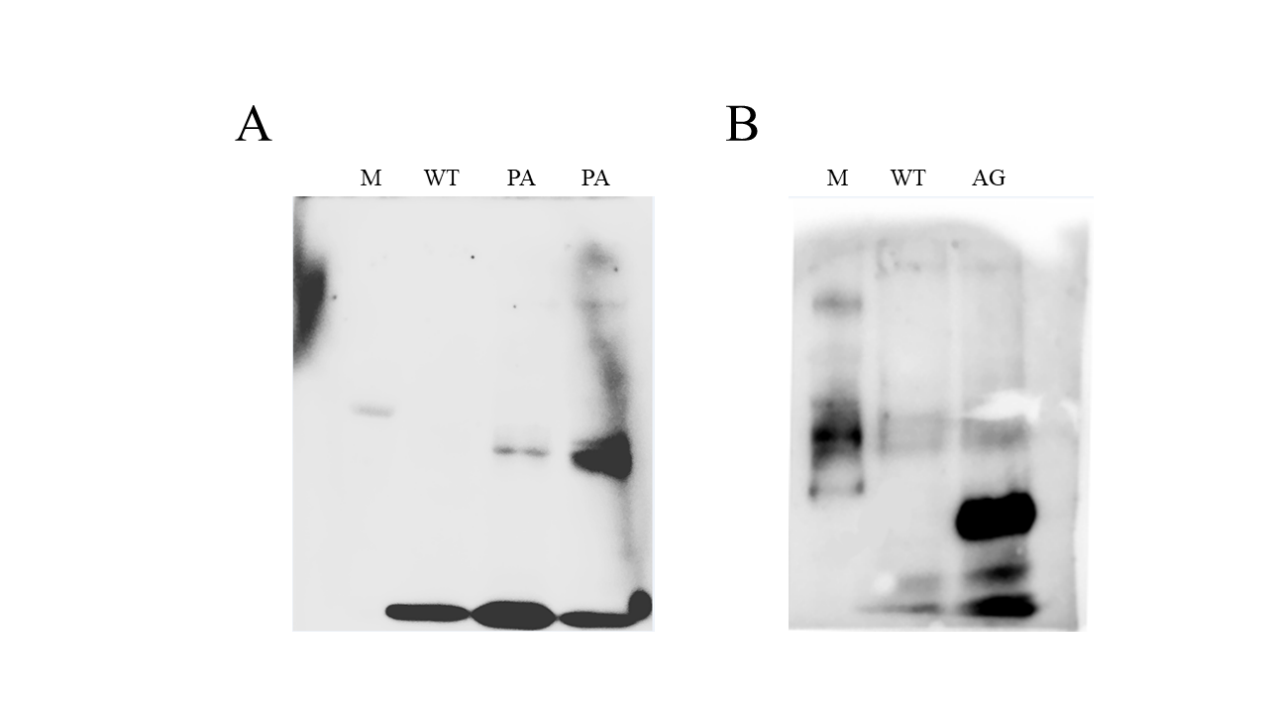


**Figure S7. The original figures of western blotting. (A)** The image of western blotting from PA strains**.** Two broods of PA were obtained and their cocoon proteins were subjected to Western blot analyses. The PA corresponding to the left lane was used for data acquisition and analysis in this study. **(B)** The image of western blotting from AG strains**.**


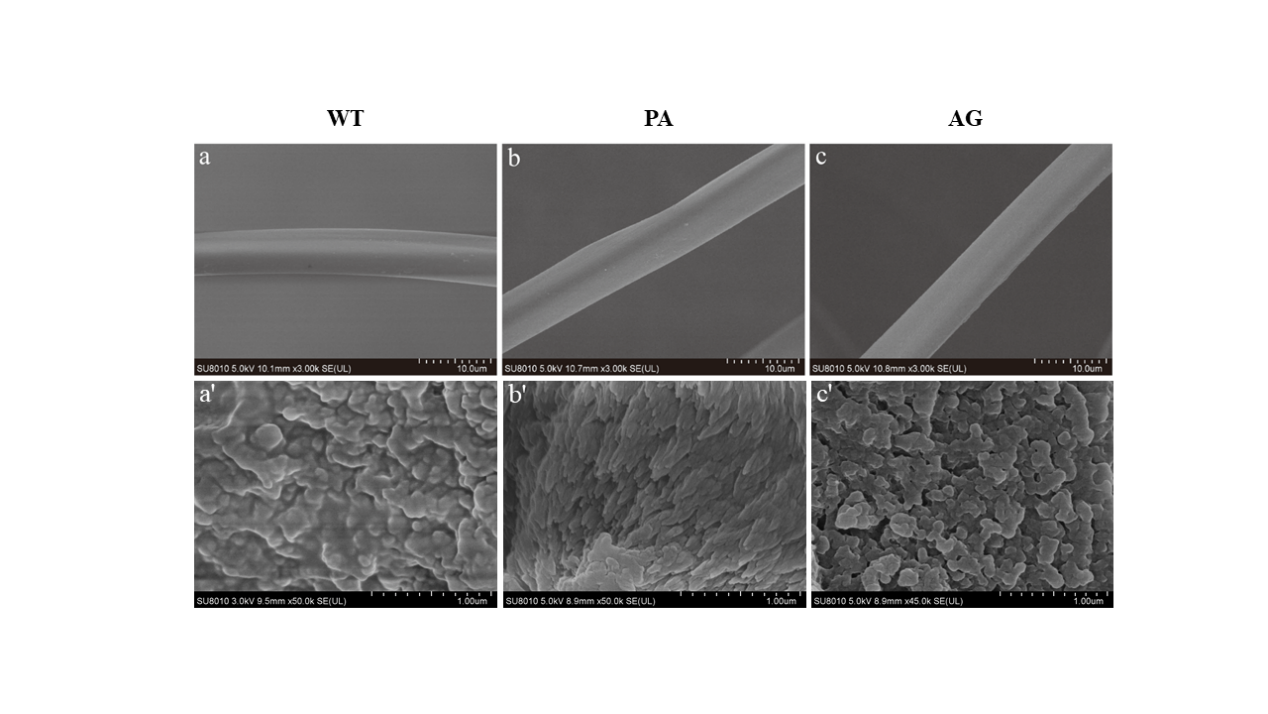


**Figure S8. Field emission scanning electron micrographs of the fibres.** The surface and cross section structures of the silk fibres derived from the WT (a, a’), PA (b, b’) and AG (c, c’).

**Table S1. The primers used for inverse PCR.**

| **Primer name** | **Sequence: 5’-3’** | **Length of the products (bp)** |
| --- | --- | --- |
| L1-F | GACAAGCACGCCTCAGCC | dependent on the inverse site |
| L1-R | TGAGTCAAAATGACGCATGATTATC |  |
| L2-F | GCTCCAAGCGGCGACTG | dependent on the inverse site |
| L2-R | GGGATGTTCTTTAGACGATGAGC |  |

**Table S2. The primers used for qRT-PCR.**

| **Primer name** | **Sequence: 5’-3’** | **Length of the products (bp)** |
| --- | --- | --- |
| q PA-F | GTGTGAATTGCACCGGTC | 72 |
| q PA-R | CGCTTGATTGCCTAGGCT |  |
| q A1-F | GTGTGAATTGCACCGGTC | 71 |
| q A1-R | TTTACCCGTCCTAGGCTT |  |
| GAPDH-F | GAAAAGGGAGCTCAAGTGGTCGC | 133 |
| GAPDH-R | CAACAAGGAATCCATCCTGAACCTC |  |

**Table S3.** **Mechanical Properties of the reconstituted silk fibers.**
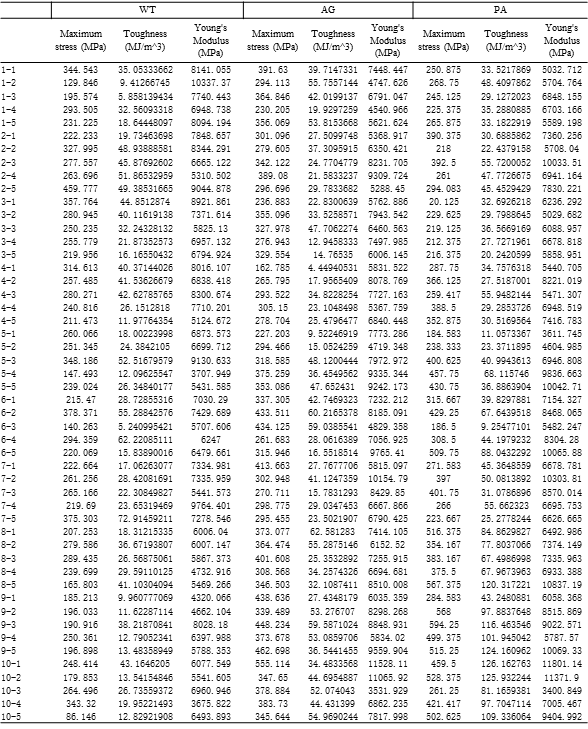


*Note: 1-1 refers to the first filament of the first cocoon.* *10-5 refers to the fifth filament of the tenth cocoon.*
